# The healthcare costs of increased body mass index–evidence from The Trøndelag Health Study

**DOI:** 10.1186/s13561-024-00512-8

**Published:** 2024-06-01

**Authors:** Christina Hansen Edwards, Johan Håkon Bjørngaard, Jonas Minet Kinge, Gunnhild Åberge Vie, Vidar Halsteinli, Rønnaug Ødegård, Bård Kulseng, Gudrun Waaler Bjørnelv

**Affiliations:** 1 Department of Public Health and Nursing, Norwegian University of Science and Technology, Trondheim, Norway; 2 Faculty of Nursing and Health Sciences, Nord University, Levanger, Norway; 3 Norwegian Institute of Public Health, Oslo, Norway; 4 Department of Health Management and Health Economics, Institute of Health and Society, University of Oslo, Oslo, Norway; 5 Regional Center for Healthcare Improvement, St. Olavs Hospital, Trondheim University Hospital, Trondheim, Norway; 6 Regional Center for Obesity Research and Innovation, Department of Surgery, St. Olavs Hospital, Trondheim, Norway; 7 Department of Clinical Molecular Medicine, Norwegian University of Science and Technology, Trondheim, Norway

**Keywords:** Obesity, Instrumental variables, Regression models, Estimation, Health, Mendelian randomization, Causal inference

## Abstract

**Background:**

Earlier studies have estimated the impact of increased body mass index (BMI) on healthcare costs. Various methods have been used to avoid potential biases and inconsistencies. Each of these methods measure different local effects and have different strengths and weaknesses.

**Methods:**

In the current study we estimate the impact of increased BMI on healthcare costs using nine common methods from the literature: multivariable regression analyses (ordinary least squares, generalized linear models, and two-part models), and instrumental variable models (using previously measured BMI, offspring BMI, and three different weighted genetic risk scores as instruments for BMI). We stratified by sex, investigated the implications of confounder adjustment, and modelled both linear and non-linear associations.

**Results:**

There was a positive effect of increased BMI in both males and females in each approach. The cost of elevated BMI was higher in models that, to a greater extent, account for endogenous relations.

**Conclusion:**

The study provides solid evidence that there is an association between BMI and healthcare costs, and demonstrates the importance of triangulation.

**Supplementary Information:**

The online version contains supplementary material available at 10.1186/s13561-024-00512-8.

## Introduction

There is a wide consensus that there is a positive association between body mass index (BMI) and healthcare costs. However, the size of the estimated effect varies substantially between studies [1–4]. For instance, in a review of the literature Kent et al. (2017) found healthcare costs to be 25–54% higher for individuals with obesity, compared to individuals with normal BMI. This variation is considerable and is likely a result of variation in methods, sample populations, and, in the case of instrumental variable analysis (IV), variations in the instrument.

A central challenge shared by researchers is that BMI is associated with, but not necessarily the cause of, a wide range of medical conditions [5]. When estimating the impact of elevated levels of BMI on healthcare cost there are several potential sources of bias: First, there may be unobserved variables influencing obesity and healthcare costs. For example, personality characteristics and time preferences. Second, there may be simultaneity bias, in that healthcare utilization might affect BMI. For instance, BMI is associated with a range of diseases, for which the pharmaceutical treatment may be obesogenic [6]. Lastly, measurement error might influence effect estimates. For instance, self-reported healthcare utilization is subject to recall bias [7, 8], and respondents typically overestimate height and underestimate weight when self-reporting [9, 10].

As obesity is a major and growing public health challenge with vast economic implications, researchers have tried to deal with the abovementioned issues by applying a variety of analytical approaches. For example, multivariable regression models, which are the most common, allow for inclusion of different sets of covariates to account for measured confounding. While IV approaches can, if using an appropriate instrument, reduce bias from both omitted variables and simultaneity [11, 12], though valid instruments have been hard to find.

The main aim of the current study was to estimate the impact of increased BMI on healthcare costs when using current and commonly used analytical approaches on the same population. By using a consistent study population, our approach allowed us to disentangle variation in effect estimates caused by different analytical approaches from variation caused by differences in the populations under analysis. We used the following analytical approaches: (i) ordinary least squares (OLS), (ii) generalized linear models (GLM), (iii) two-part models (2PM), (iv) time-lagged IV models using previously measured BMI as instruments for current BMI, (v) offspring IV models, using BMI of the oldest offspring available as an instrument for parental BMI, and vi-viii) IV models using three different genetic risk scores (GRS) as instruments for BMI (Mendelian randomization).

The GLM and 2PM approaches used in this paper are examples of multivariable models that are frequently used, and are able to handle cost data well [13]. Kim and Basu [2], compared multivariable models on the same sample with different age-stratifications, and with adjustment for different sets of covariates. They found that costs did not differ substantially between the models used, were higher in the sample with individuals aged 65 years and above and were reduced when adjusting for all covariates and obesity-related diseases.

Time-lagged IV models that use past BMI as an instrument for future BMI are used to account for some aspects of simultaneity. For example, obesity may increase the risk of cancer in an individual, and thereby increase healthcare costs. At the same time, however, cancer may have led to *cachexia* (a disorder causing muscle and weight loss), which is associated with both obesity and healthcare costs [14]. Using lagged BMI as an instrument is potentially problematic as the instrument might be correlated with the outcome variable directly. For example, past BMI might be correlated with future healthcare utilization due to strain on the musculoskeletal system.

Offspring BMI serves well as an instrument for parental BMI because offspring BMI is arguably independent of parental healthcare costs, yet strongly correlated with parental BMI due to high heritability [15]. As a result, these analyses provide estimates that are less likely to be biased by simultaneity or omitted variables. While there are few obvious concerns with these models, one possible limitation is that the instrument might affect the outcome variable directly or indirectly for instance through in utero effects and/or the effects of sharing a household environment might impact both BMI and healthcare utilization [16–18]. Another challenge, when comparing results from different approaches, is that these models require data on parent-offspring pairs, which is a sub-sample of the data.

The last approach was to use three different genetic risk scores (GRS) constructed from genetic variants that have been found to be associated with BMI in genome wide association studies as instruments for BMI. Although genes are randomly allocated from parents to offspring at conception, there are some challenges. First, the use of GRS as IVs is problematic if the genetic variants in the GRS influence the outcome. The latter might be the case due to population stratification (differences in allele frequencies in subpopulations), assortative mating (i.e. individuals with similar characteristics tend to mate with each other), linkage disequilibrium (i.e. genetic variants that are close together in a gene are correlated), and horizontal pleiotropy (i.e. that one genetic variant affects multiple traits) [19–21]. Although methods have been proposed to identify, and to some extent deal with, these issues, there is still a lot to be understood about the use of genetic variants as instruments [22, 23].

When we compare the different analytical approaches, we find that healthcare cost estimates are sensitive to the analytical approach used, but insensitive to inclusion of covariates. In the main set of analyses, the effect estimates when using the different analytical approaches varied from $70 to $363 for males and from $59 to $121 for females. Except for the IV analyses using offspring BMI as an instrument for males, the confidence intervals of the effects estimated from the different analytical approaches were overlapping. For males, the inclusion of covariates led to increased ($13 to $22) effect estimates in the multivariable models, and decreased ($-3 - ¤-42) in the IV-models. For females, the effect estimates decreased (by $2 to $42) in all the models.

This paper will proceed as follows: In the next section (Data) we present the data sources used for our study. In the section entitled Analytical approaches, we describe the analytical approaches used. In the Results section we present the main findings, and in the Discussion and conclusion section we provide a conclusion and discussion of our findings.

## Data

Using an 11-digit personal identification number, we linked individual-level data from three sources: The Nord Trøndelag Health Studies (HUNT), the Norwegian Patient Register (NPR) and Statistics Norway.

The HUNT study is a longitudinal population-based health study. All citizens living in the former county of Nord-Trøndelag, aged > 20 years, were invited to participate. We used data from three waves of the study: HUNT 1 (performed in the years 1984–1986, participation rate: 88%), HUNT 2 (performed in the years 1995–1997, participation rate: 69%), and HUNT 3 (performed in the years 2006–2008, participation rate: 54%). All together $\sim$122 000 individuals participated. In each round information was gathered from self-completed questionnaires, clinical measurements (height and weight) and biological samples (genetic information). HUNT is considered largely representative of the Norwegian population with regards to geography, economy, industry, sources of income, age distribution, morbidity, and mortality [24].

All hospitals in Norway report patient activity electronically to NPR as part of their funding scheme (activity-based-funding). Thus, NPR includes information on all specialist care provided in Norway, which includes somatic (i.e. physical/physiological aspects of the body) and psychiatric inpatient-, day-, and outpatient- care. The electronic reports include information about patients’ diagnoses (ICD-10 codes) and treatments/procedures used to diagnose/treat the patient. This information forms the basis for grouping all patient episodes into one of approximately 900 diagnosis related groups (DRGs). Each DRG holds an associated cost-weight that can be used to estimate the average cost of an inpatient-, day-, or outpatient- care episode. The DRG-costs are expected to cover the average costs of a treatment including complications during a hospital stay, within a DRG-group, in Norway.

Lastly, data from Statistics Norway (SSB) provided information on sociodemographic characteristics such as income, education, country of birth and living status (i.e., whether individuals per 1st January, each year, were resident in Norway, living abroad or dead, and the date of change from previous status).

### Calculation of healthcare costs

We estimated healthcare costs in the period 2009–2016. Healthcare costs were estimated as Norwegian Kroner (NOK) 2016 and inflated based on the Norwegian consumer price index [25]. We converted healthcare costs from NOK to US dollars (USD) using the average exchange rate during 2016 (1 USD = 8.3987 NOK) [26]. We estimated an average healthcare cost per year in the period 2009–2016. First, we estimated the average cost per year (i.e., the average cost in 2009, the average cost in 2010 etc.). In these estimates, we only used data from individuals who had been residing in Norway for the entire year under analysis (i.e., for the costing year 2009, individuals were included if they were alive January 1st, 2010, and if they had lived in Norway for the whole year, that is January 1st 2009 – January 1st 2010). Those who died during the year were included, while those who were not living in Norway were set as missing. Individuals who were set as missing because they moved to/from Norway during 2009 could appear in later costing years, given the same premises as listed above. Average annual healthcare costs were calculated as the sum of costs for each year (2009–2016) for each individual, divided by the number of years that the individual was alive and residing in Norway (and thereby had the potential to incur costs)^1^. This approach for computation of healthcare costs implies that we do not capture BMI-specific variations in costs for the same type of treatment. However, it is likely that for some treatments the cost might depend on BMI. For instance, for some surgical procedures, the duration of surgery might be longer for patients with obesity [27].

### Body mass index

We used BMI as a measure of obesity in our study. Information on height and weight was measured by a research assistant as part of the examination in the HUNT studies. Weight was measured to the nearest half kilogram while participants were wearing light clothes and no shoes. Height was measured to the nearest centimeter [28]. We estimated BMI by dividing each participant’s weight (in kilograms, kg) by their height (in meters, m) squared: (kg/m^2^). In all the analyses, BMI was included as a continuous variable. In the results we occasionally refer to different classes of obesity, and here we have used the World Health Organization’s cut-offs)^2^. BMI has been criticized for not accounting for the bodily distribution of fat, or body composition, but is also a simple and commonly used measure, allowing for comparison between studies [29].

### Covariates

Relevant covariates were selected based on the findings from a literature review of studies investigating the association between BMI and healthcare costs by Kent, Fusco [1]. We assessed all the covariates listed by Kent, Fusco [1] and included the following covariates in the fully adjusted models: education, smoking status, marital status, resident in rural or urban area, income, and country of birth.

Age was measured continuously. We also tested the use of age squared in all analyses. Education was categorized as [1] primary school [2], secondary school [3], higher education (short, ≤ 3 years), and [3] higher education (long, ≥ 3 years) (SSB). Smoking status was categorized as [1] never smokers [2], previous smokers [3], daily smokers and [4] occasional smokers. Marital status was categorized as [1] married or cohabitants [2], never married [3], widows/ widowers, or [4] divorced or separated (SSB). Urbanity (categorized as [1] urban/ [2] rural) was defined as those living in one of the five municipalities in former Nord-Trøndelag County that were considered densely populated urban areas with town-status^3^ at the time of invitation to the HUNT Study [30]. Income was measured continuously at the individual level, as gross wage income. Country of birth was categorized as [1] those born in Norway from Norwegian parents and [2] all others^4^.

## Analytical approaches

Sex-specific effects of BMI on healthcare costs were estimated using several analytical approaches: multivariable models, time-lagged IV models, offspring IV models, and GRS IV models.

**Fig. 1:**
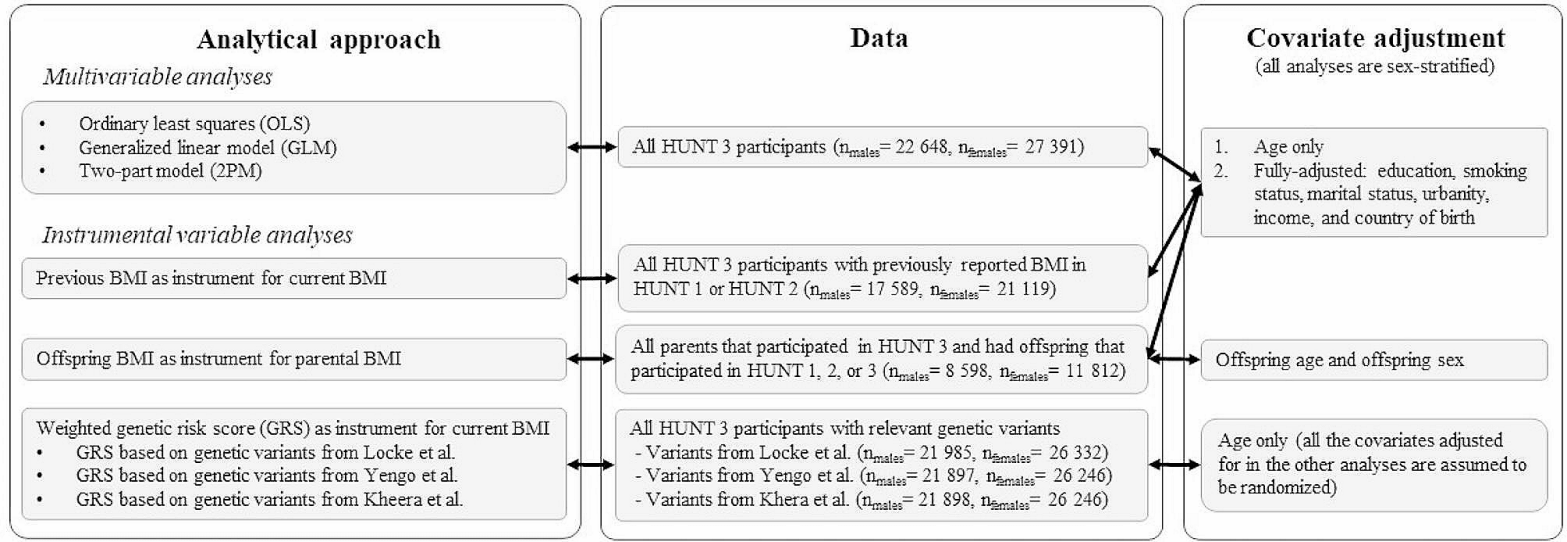
Overview of estimation strategies, data used, and covariate adjustment. All studies included only individuals where height and weight had been measured (this information was available for 99% of participants in HUNT 3)

The analytical approaches required inclusion of different samples. Figure 1 provides an overview of the sex-specific sample sizes and covariates included for each of the analytical approaches applied. The sample for the GRS IV models included all participants with height and weight recorded in HUNT 3 and information about relevant BMI-specific genetic variants. We report descriptive statistics for all samples to assess if key characteristics of the samples change as participants are included/excluded and conduct analyses across samples.

For all the models (except the GRS IV studies) we ran age-adjusted models, and fully adjusted models (in which we adjusted for: age, education, smoking status, marital status, urbanity, income, and country of birth). In the GRS IV studies, we assume that the genetic variants included in the GRS are independent of sociodemographic confounders and therefore we only adjust for age (and refer to these models as fully-adjusted). Since the association between BMI and healthcare costs has previously been found to be non-linear [31], we assessed both the linear and the non-linear relationship between BMI and healthcare costs in all analytical approaches. Non-linearity was only assessed in the fully-adjusted models, and reported as the marginal effect of increasing BMI by one BMI-point.

### Multivariable regression analyses

We conducted analyses using three different multivariable regression models that have frequently been used in previous studies to estimate the effect of BMI on healthcare costs: ordinary least squares (OLS), generalized linear models (GLM) and two-part models (2PM).

The GLM and 2PM were specified in accordance with commonly used [1, 2] and recommended practices [13]; for the GLM we used a log-link and a gamma distribution, and for the 2PM we used a logistic regression for the first part of the model, and a GLM with a log-link and a gamma distribution for the second part of the model. To provide a single estimate for the association between BMI and healthcare costs, we combined the two parts of the 2PM into one estimate using the methods described by Deb and coworkers (2017) [13]. To allow for investigation of non-linear effects, a squared BMI-term was included in the regression equations.

To avoid time-lag in our analyses, we restricted our multivariable analyses to HUNT 3 participants (*n* = 50,410), Fig. 1. A time-lag would have occurred if we included individuals from HUNT 2 (1990s) or HUNT 1 (1980s) due to the time interval between the years that HUNT 2 and HUNT 1 were conducted, and the years for which data on healthcare costs were available (2009–2016).

### Instrumental variable (IV) analyses

**Fig. 2:**
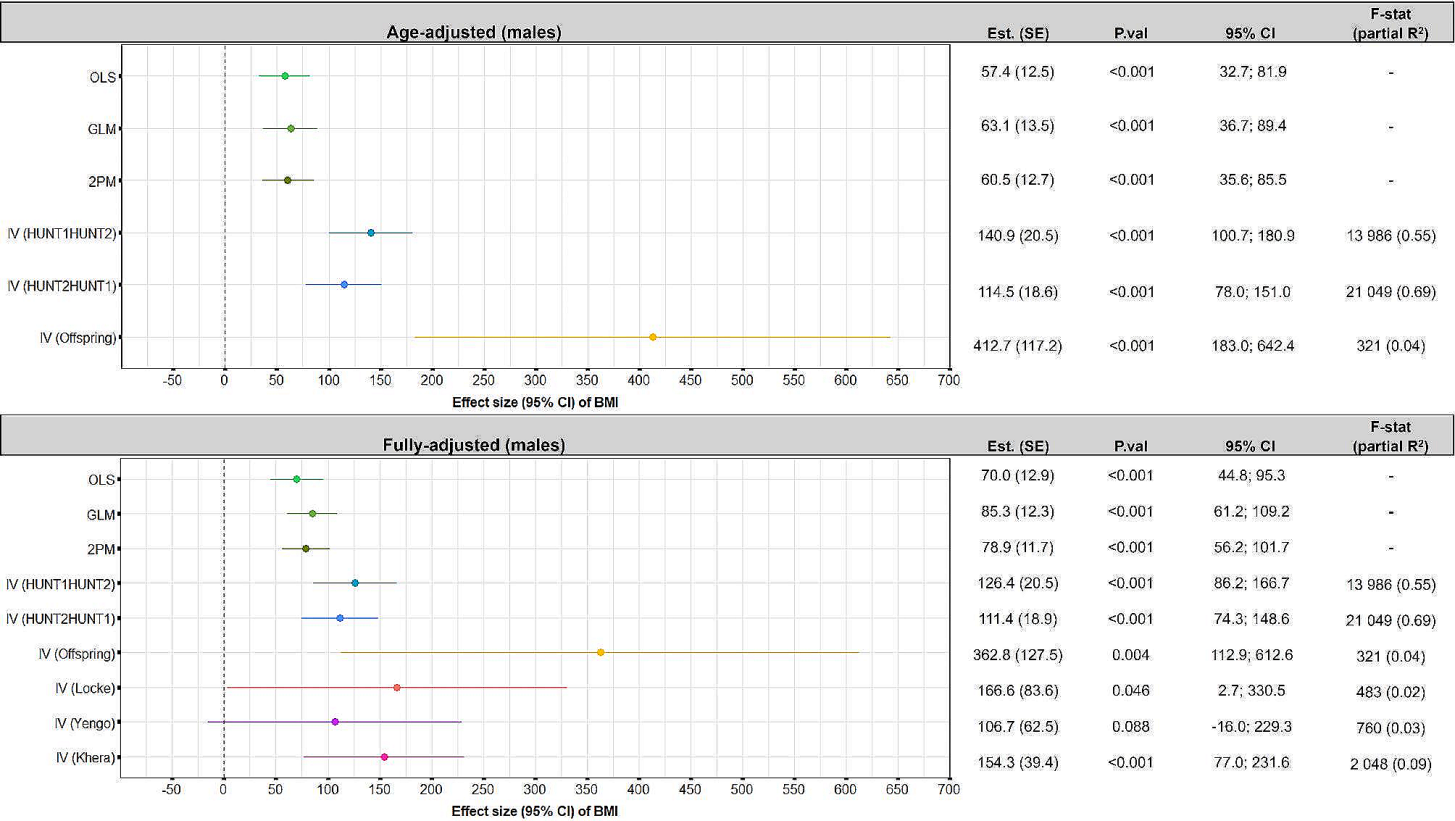
Effect and 95% confidence intervals of BMI on healthcare costs using different analytical approaches, for males, with adjustment for age (top panel), and fully-adjusted models (bottom panel). In the fully adjusted analyses the OLS, GLM, 2PM, lagged IV-analyses, and the offspring IV-analyses, estimates were adjusted for age, education, smoking status, marital status, resident in rural or urban area, income and country of birth. In the offspring IV-analyses we also adjusted for offspring age and sex, and in the GRS based IV-analyses we only adjusted for age (since a prerequisite for these models is that there is no need for further adjustment because our genes are randomly assigned at conception, and therefore assumed to be uncorrelated with confounding factors)

**Fig. 3:**
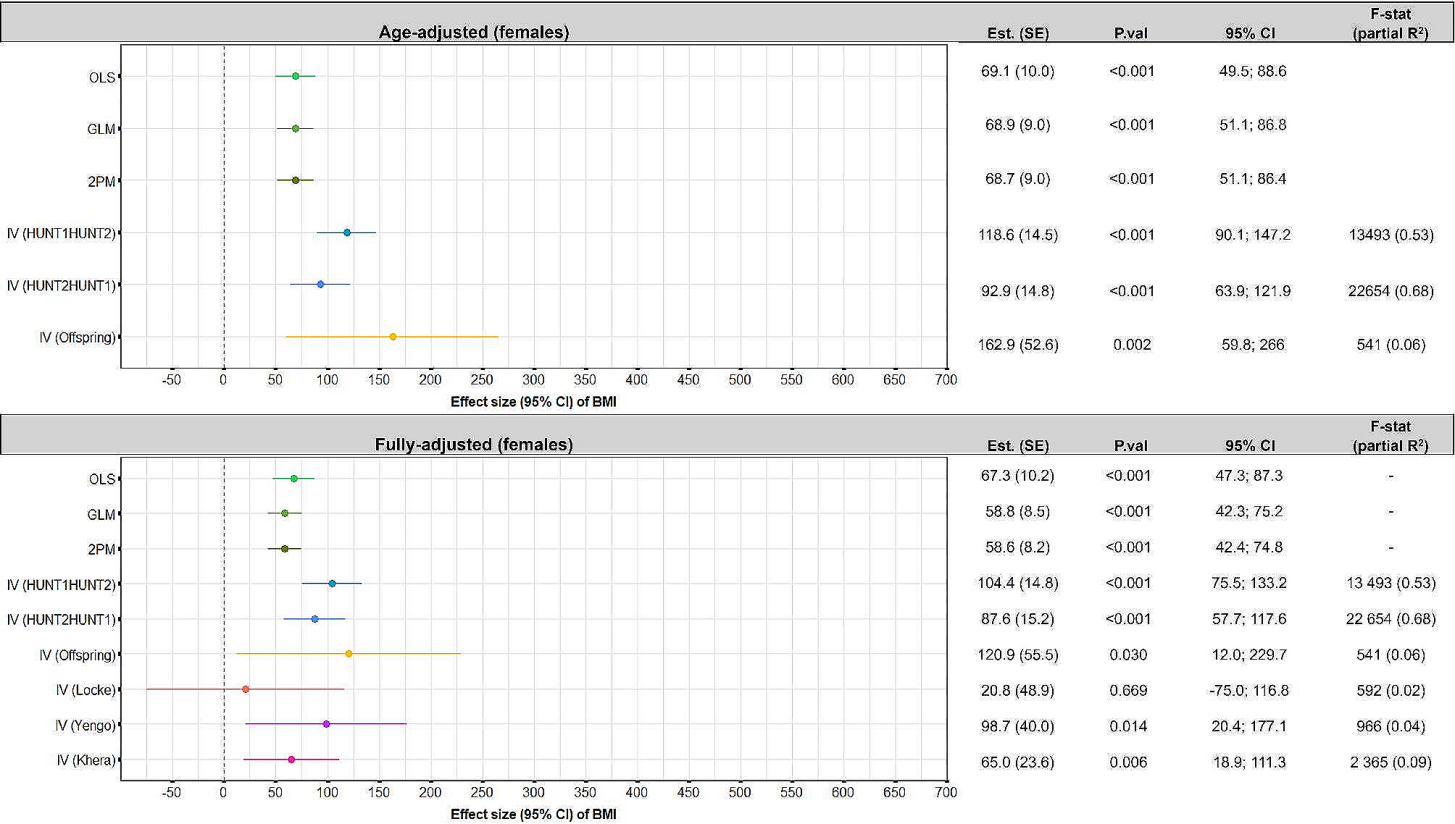
Effect and 95% confidence intervals of BMI on healthcare costs using different analytical approaches, for females, with adjustment for age (top panel), and fully-adjusted models (bottom panel). In the fully adjusted analyses the OLS, GLM, 2PM, lagged IV-analyses, and the offspring IV-analyses, estimates were adjusted for age, education, smoking status, marital status, resident in rural or urban area, income and country of birth. In the offspring IV-analyses we also adjusted for offspring age and sex, and in the GRS based IV-analyses we only adjusted for age (since a prerequisite for these models is that there is no need for further adjustment because our genes are randomly assigned at conception, and therefore assumed to be uncorrelated with confounding factors)

IV models can provide causal estimates of the effect of BMI on healthcare costs, provided that the underlying IV-assumptions are satisfied. In brief, the following assumptions must be satisfied: (1) The instrument must be correlated with the exposure variable (BMI) (2) There should not be any omitted variables influencing the association between the instrument and the outcome (healthcare costs) (3) The instrument must be related to the outcome (here healthcare costs), only via its effect on the exposure (BMI). For each of the IV analyses we report the F-statistics and partial R^2^ which indicates the strength of the instrument (F) and how much of the variation in the exposure that the instrument explains (partial R^2^) (Figs. 2 and 3). We used three different types of instruments in the IV-models: previous BMI, offspring BMI, and weighted genetics risk scores (GRS):

### Previous BMI as instrument for current BMI (time-lag IV)

We ran two-stage least squares (2SLS) IV models using previous BMI as instruments for current BMI. The time-gap between HUNT 1 and HUNT 3 is 20–24 years, and the time-gap between HUNT 2 and HUNT 3 is 9–13 years. Respondents were included in the analyses if height and weight were measured in HUNT 3 and HUNT 1 and/or HUNT 2 (Fig. 1). We conducted two separate analyses; one, where the BMI in HUNT 3 was instrumented by BMI in HUNT 1 (when available), and otherwise instrumented by BMI in HUNT 2 (IV HUNT 1| HUNT 2). Another, where the BMI in HUNT 3 was instrumented by BMI in HUNT 2 (when available), and otherwise instrumented by BMI in HUNT 1 (IV HUNT 2| HUNT 1).

In each of the time-lagged IV analyses, we estimated the age-adjusted relationship between BMI and healthcare costs, and subsequently the relationship with full covariate adjustment (Fig. 1). To allow for investigation of non-linear effects, a squared BMI-term was included in the 2SLS; a squared-term of current BMI was instrumented by a squared-term of previous BMI.

### Offspring BMI as an instrument for parental BMI (Offspring IV-model)

We ran 2SLS IV models using the BMI of the oldest offspring that had participated in HUNT as an instrument for parental BMI. In these analyses we included all parents that had participated in HUNT 3, and that had an offspring (child) that had participated in either HUNT 1, HUNT 2 or HUNT 3 (Fig. 1). We always chose the oldest offspring, and if she or he had participated in more than one round of HUNT, we chose values from the most recent measurement point. In the offspring-model, we ran age-adjusted and fully-adjusted models, and in the first part of the model, we also adjusted for the age and sex of the offspring [32]. To allow for investigation of non-linear effects, a squared BMI-term was included in the 2SLS; a squared-term of parents BMI was instrumented by a squared-term of offspring’s BMI.

### GRS as instruments for individual BMI (Mendelian Randomization, MR)

We ran 2SLS IV models using three different GRS as instruments for BMI. For these analyses, we included all individuals with information on BMI-related genetic variants in HUNT 3 (Fig. 1). Using data on the BMI-related genetic variants we generated three weighted GRS: one score based on the variants reported by Locke, Kahali [33], one score based on the variants reported by Yengo, Sidorenko [34], and one based on the variants reported by Khera, Chaffin [35]. The Locke-score was based on 96 of the 97 genetic variants reported by Locke, Kahali [33]. The Yengo-score was based on 936 of the 941 main variants reported by Yengo, Sidorenko [34], and the Khera-score was based on ~ 2.07 million genetic variants of ~ 2.1 million genetic variants reported by Khera, Chaffin [35]. Genetic variants that were excluded from the scores were excluded due to poor imputation quality, linkage disequilibrium, or because they were not available.

The scores were constructed by multiplying the number of BMI-increasing alleles for each participant with the beta-coefficients reported in each of the abovementioned papers and adding these together for each participant. For a more thorough description of the GRS-construction, see, for example Brandkvist, Bjørngaard [36]. In these models we adjusted for age only, because all other covariates are assumed to be unrelated to the genetic variants (since these are assumed to be randomized at conception in accordance with Gregor Mendel’s Principle of Independent Assortment [37]). We refer to these models as fully adjusted. To allow for investigation of non-linear effects in these analyses, we used the semi-parametric methods proposed by Staley and Burgess [38].

## Results

### Descriptive statistics

Our sample consisted of 22 648 males and 27 391 females. The majority had overweight (males: 53%; females: 38%), while 19% of males and 17% of females had class 1 obesity, and 4% of males and 7% of females had class 2 obesity. Less than 1% of males and females had underweight. The mean age was 53 years (and the SD 19) for both males and females. The majority had completed secondary school (males: 58%; females: 48%), were never smokers (males: 40%; females: 43%), were married/cohabitants (males: 62%; females: 56%), lived in urban areas (males: 63%; females: 64%), and were born in Norway from Norwegian parents (males: 97%; females: 96%). The average healthcare costs for participants of HUNT 3 were $2 834 (SD 6742) for males, and $2 652 (SD 6 165) for females.

**Table 1:** Descriptive sample information

Variable	Category	Males *n* (%)			Females *n* (%)	
Total	All	22 648	(100.0)		27 391	(100.0)
BMI-category^a^	Underweight	69	(0.3)		234	(0.9)
	Normal weight	5 631	(24.9)		10 418	(38.0)
	Overweight	11 863	(52.4)		10 334	(37.7)
	Class 1 obesity	4 241	(18.7)		4 599	(16.8)
	Class 2 obesity	844	(3.7)		1 806	(6.6)
Age - category	19–29 years	1 840	(8.1)		1 840	(6.7)
	30–39 years	2 837	(12.5)		3 985	(14.5)
	40–49 years	4 536	(20.0)		5 418	(19.8)
	50–59 years	5 371	(23.7)		5 960	(21.8)
	60–69 years	4 593	(20.3)		5 072	(18.5)
	70–79 years	2 569	(11.3)		3 026	(11.0)
	80–89 years	861	(3.8)		1 231	(4.5)
	90 + years	41	(0.2)		93	(0.3)
	Missing	0	(0.0)		0	(0.0)
Educational level	Primary School	4 524	(20.0)		6 390	(23.3)
	Secondary School	13 091	(57.8)		13 098	(47.8)
	Higher education, short	3 626	(16.0)		7 046	(25.7)
	Higher education, long	1 347	(5.9)		737	(2.7)
	Missing	60	(0.3)		120	(0.4)
Smoking Status	Never smoker	8 970	(39.6)		11 829	(43.2)
	Past smoker	7 976	(35.2)		7 863	(28.7)
	Daily smoker	3 313	(14.6)		5 133	(18.7)
	Occasional smoker	1 820	(8.0)		1 757	(6.4)
	Missing	569	(2.5)		809	(3.0)
Marital status	Married / cohabitants	13 994	(61.8)		15 372	(56.1)
	Unmarried	5 761	(25.4)		5 826	(21.3)
	Widow/widower	766	(3.4)		3 255	(11.9)
	Divorced/Separated	2 087	(9.2)		2 899	(10.6)
	Missing	40	(0.2)		39	(0.1)
Urbanity	Urban	14 337	(63.3)		17 420	(63.6)
	Rural	8 049	(35.5)		9 573	(34.9)
	Missing	262	(1.2)		398	(1.5)
Immigration status	Born in Norway	21 899	(96.7)		26 303	(96.0)
	Born elsewhere	749	(3.3)		1 088	(4.0)
	Missing	0	(0.0)		0	(0.0)

a = BMI-categories following WHO categorization: underweight (BMI < 18.5 kg/m^2^), normal weight (BMI 18.5–25 kg/m^2^), overweight (BMI 25–30 kg/m^2^), class 1 obesity (BMI 30–35 kg/m^2^), and class 2 obesity (BMI 35–40 kg/m^2^).

Compared with the sample used for the multivariable analyses (Table 1), the sample used for the IV analyses with time-lag were older, and a greater proportion were married (and fewer unmarried) (Supplementary Table S1, Supplementary Figure S1 and S2). The sample used for the offspring analyses differed the most from the sample used for the multivariable analyses (Supplementary Table S2, Supplementary Figure S1 and S2). In the offspring sample, the respondents were older, and the level of education was lower, particularly among females. In addition, there were fewer never smokers and more past smokers (especially among males), and there were more married participants and fewer unmarried as well as more widowers/widows. The characteristics of the sample used for the IV analyses using GRS were highly comparable to those of the sample used for the multivariable analyses (Supplementary Table S3, Supplementary Figure S1 and S2).

### Average effect of BMI on healthcare costs

BMI was positively associated with healthcare costs for both males (Fig. 2) and females (Fig. 3). This finding was consistent across analytical approaches and with inclusion of different sets of covariates. Overall, there was a tendency that the costs estimated from the multivariable models were lower and more precise than the costs estimated from the IV models.

Inclusion of the full set of covariates had different effects for males and females. For males, the effect estimates increased in all the multivariable models (by: $12.6 (OLS), $22.2 (GLM), and $18.4 (2PM)), while the effect estimate was lower in the time-lag IV (HUNT1|HUNT2) (by: $-14.5), in the time-lag IV (HUNT2|HUNT1) (by -$3.1), and in the offspring IV model (by: $-49.9). For females the effect estimates were smaller in all models when all covariates were included in the models (by: $-1.8 (OLS), $-10.1 (GLM and 2PM), $-14.2 (time-lag IV (HUNT1|HUNT2), $-5.3 (time-lag IV (HUNT2|HUNT1), and $-42 (offspring IV)).

### Results of age-adjusted analyses

For males, a unit increase in BMI increased the average annual healthcare costs by $57.4 – $63.1 in the multivariable models, and by $114.5 – $412.7 in the IV-models (*p* < 0.01). For females, a unit increase in BMI increased average annual healthcare costs by $68.7 – $69.1 in the multivariable models, and by $92.9 – $162.9 in the IV models (p-value < 0.01 in all the analyses).

### Results from fully-adjusted models

In the multivariable models, a unit increase in BMI increased average costs by $70.0 – $85.3 for males, and by $58.6 – $67.3 for females. In the fully adjusted time-lag and offspring IV models, the average estimated cost of a unit increase in BMI varied from $111.4 – $362.8 for males and $87.6 – $120.9 for females, when using the different models. In the GRS IV models, the average estimated costs increased by $106.7 – $166.6 for males, and by $20.8 – $98.7 for females, per unit increase in BMI. The coefficient estimates of the control variables in each analytical approach had overlapping confidence intervals. The only exception was that in the offspring IV for males the coefficient for the category *unmarried* was significantly lower than in the rest of the analyses (Supplementary information Figure S3 and S4).

### Comparison of data samples

Since the offspring-sample was markedly different from the main sample for some characteristics, we conducted a sensitivity analysis where we ran all the fully-adjusted analyses on both samples (Supplementary information Figure S5). This was done to assess the effect of these differences in sample characteristics, on effects estimated from different analytical approaches. The confidence intervals of the effect estimates were largely overlapping in all the analyses. In most (all except time-lag IV (HUNT2|HUNT1 for females, and IV (Yengo) for males) of the analyses, the offspring sample resulted in slightly higher cost estimates.

### Non-linear analyses

**Fig. 4:**
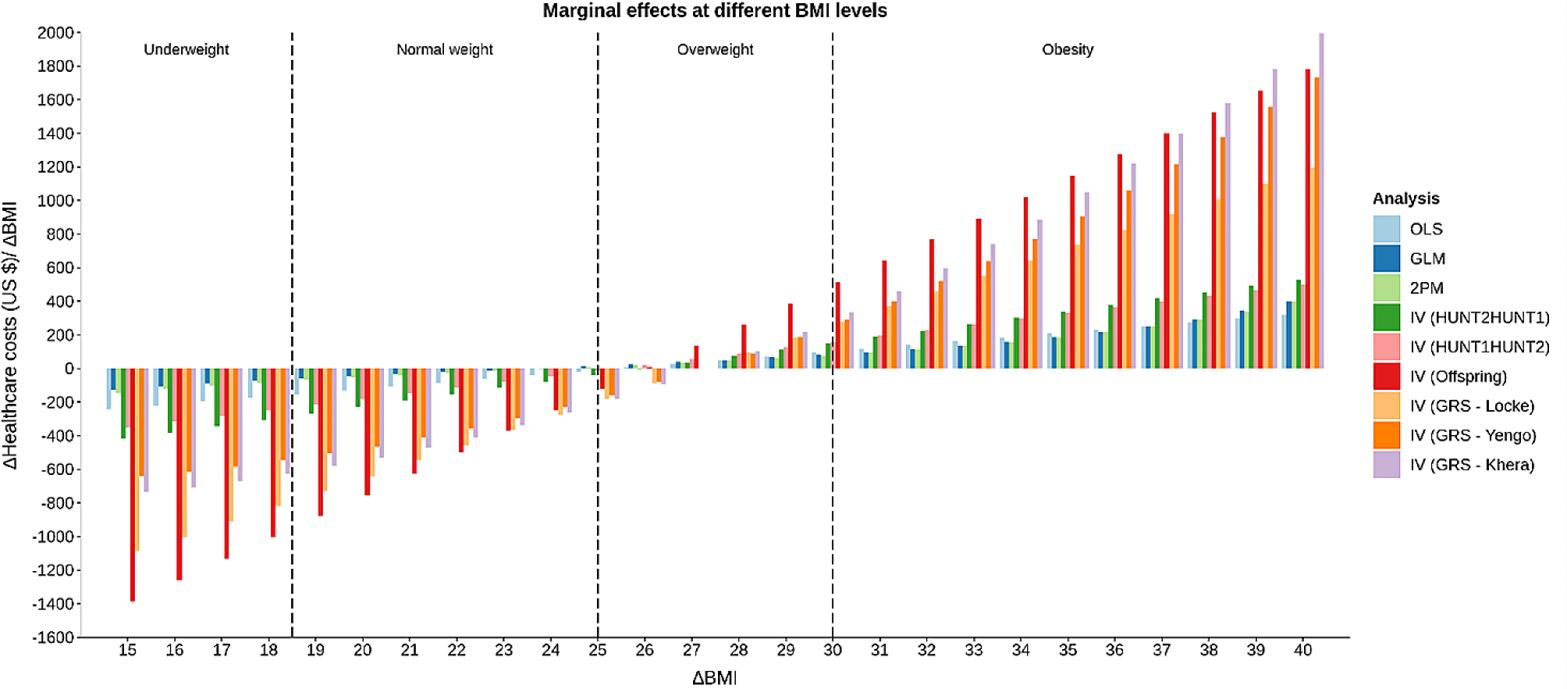
Results of non-linear analyses for males, showing the marginal effect (increasing healthcare costs) of a *one-unit* increase in BMI, using the different analytical approaches

**Fig. 5:**
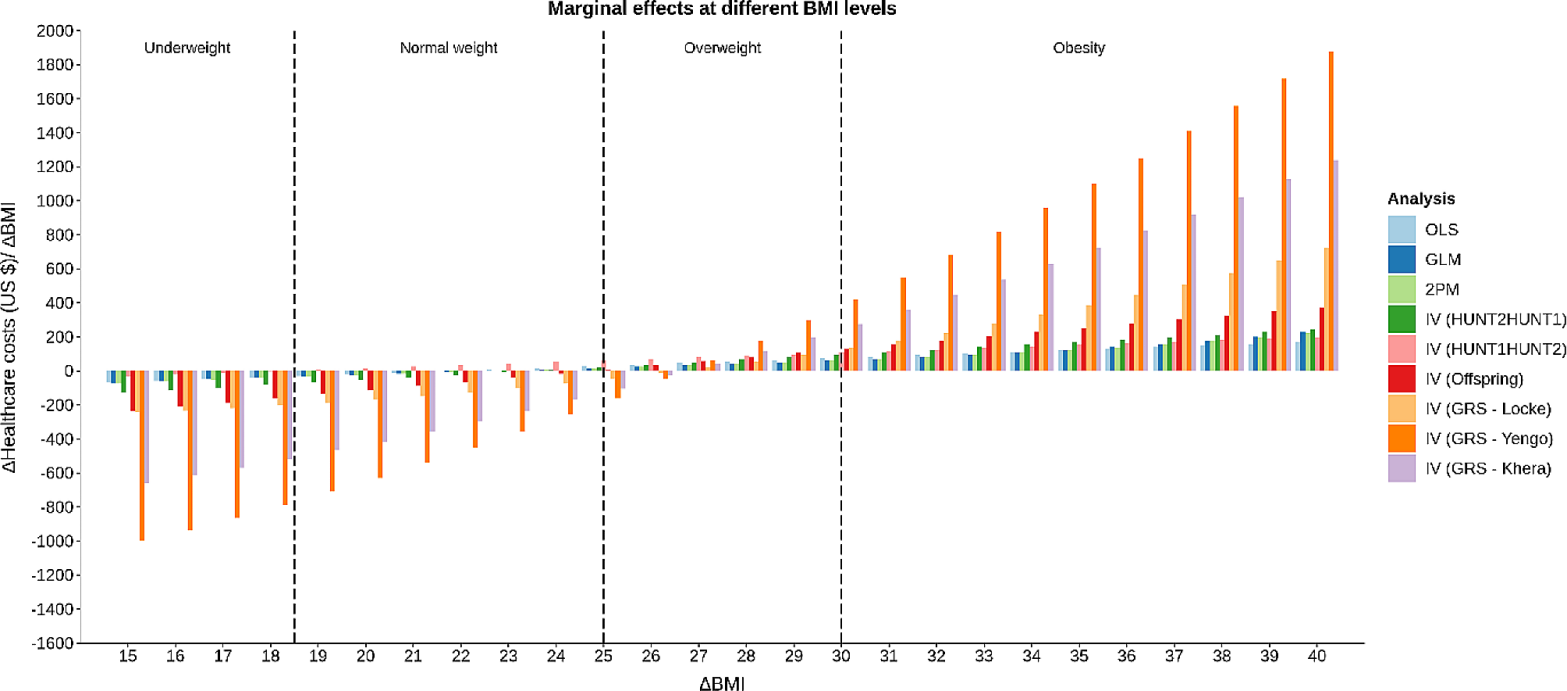
Results of non-linear analyses for females, showing the marginal effect (increasing healthcare costs) of a one-unit increase in BMI, using the different analytical approaches

There was evidence of non-linearity in the analyses for both males (Fig. 4) and females (Fig. 5). The non-linear tendencies were, however, more prominent among males. The marginal effects of increasing an individuals’ BMI by one BMI-point was negative for low BMI-levels and positive at higher BMI-levels indicating a u-shaped relationship between healthcare costs and BMI. Marginal effects went from negative to positive in the normal weight/ overweight range (BMI 25–26) for males and in the normal weight range (BMI 19–26) for females. The non-linear curves estimated using different analytical approaches were similar, but when IV models were used the curves became steeper.

The marginal cost of increasing BMI with one BMI-point was largest at the extremes of the BMI distribution (Figs. 4 and 5). For example, healthcare costs were estimated to increase more, on average, for individuals moving from BMI 39 to BMI 40 than for individuals moving from BMI 29 to BMI 30. The size of the effect estimates also varied quite considerably between the analytical approach used.

## Discussion and conclusion

There was a positive effect of increased BMI in both males and females. Although the precision and size of the coefficient varied across the models, each were of the same sign. The 95% confidence intervals tended to overlap between the analytical approaches, however there were some trends in the effect sizes. Our findings suggest that the costs of elevated BMI increased further when using methods that to a larger extent account for bias from simultaneity and omitted variables. This is in line with findings of past studies [1–4], but in those studies, it was not clear whether this was due to the analytical approach, characteristics of the study sample, or the study setting. Our study also provides solid evidence that increased BMI leads to higher healthcare costs for both males and females, and that the effect of additional units of BMI on healthcare costs increases with increasing BMI. This finding was consistent across the range of analytical approaches used and when different covariates were included in the models. Overall, the effect estimates were smaller for females than for males. In addition, the effect of adjusting for known confounders, and the effect of the analytical approach used on the effect estimates seemed to differ between the sexes.

Our results demonstrate how the choice of IV influenced the effect estimates. Previous studies have argued for the validity of genetic variants and offspring BMI as instruments for BMI [32, 39, 40]. In our analyses, the offspring-estimates stand out, as cost estimates were particularly high in these models, especially for males. This could be due to unobserved associations between offspring BMI and omitted variables or between offspring BMI and parental healthcare costs, and this should be investigated further. If we compare our offspring IV estimates with those of Cawley and Meyerhoefer [32] we notice some important differences. First, although the inflation adjusted (adjusted to 2016 price levels 1 USD 2005 = 1.24 USD in 2016 [41]) 2PM marginal effects are similar for males ($73 in their paper vs. $79 in our paper) and females ($58 in their paper vs. $59 in our paper), the offspring IV estimates are quite different. The marginal effects estimated from their offspring IV model was 1.4 times larger for males, and 3.7 times larger for females compared with the corresponding 2PM estimate. In our study, the marginal effect estimated from the linear offspring IV model was 4.6 times larger than the 2PM estimate for males, and 2.1 times larger than the 2PM estimate for females. These are large discrepancies, which cast doubt on the validity of the offspring instrument and should be explored in future studies. However, these differences could also be due to differences in sample characteristics.

The effect estimates from the GRS IV models vary depending on which genetic variants are included in the score. The estimates vary from $107–$167 for males and from $21–$99 for females, with varying precision. This too is an interesting point for further research, and future studies should aim at conducting sensitivity analyses to try to investigate possible violations of the exclusion restriction, in particular, pleiotropy (i.e., that genetic variants that are associated with BMI are also associated with other phenotypes that might influence the relationship between BMI and healthcare costs) when using the different GRS.

Although the analytical approach influences the size and precision of the effect estimates, we cannot state which models’ effect estimate is a causal estimate because each approach has its advantages and limitations. Moreover, there are aspects of the relationship between BMI and healthcare costs that we were not able to account for using our data: for example: the time lived with BMI and the BMI trajectories of participants. In this study we have focused on costs within specialist care services, thus excluding various components of the healthcare system in Norway, such as: home and nursing home care, pharmaceutical care, primary care, and private care. The extent and types of endogeneity bias may vary between care providers, thereby influencing costs. It would be of interest to conduct a similar study across all healthcare settings to obtain a more comprehensive overview of the consequences of methodological decisions.

In general, the assumptions for nonlinear instrumental variable estimations are strong, and therefore the effects from the non-linear IV studies should be interpreted with caution. Concerns have particularly been raised about methods we have used for nonlinear estimation in the IV analyses using genetic instruments [42].

The response rate in HUNT 3 was 54%, and the reasons for non-participation were: lack of time or inconvenient session (51% females, 57% males), not having received an invitation (10%), and among the oldest being too ill or not seeing the benefit of participation were also common reasons for not participating [43]. Participation was dependent on age, sex, and socioeconomic status, BMI was slightly lower (albeit self-reported) among non-responders, and depending on the health problem under study, non-responders had higher prevalence of some conditions and lower prevalence of others [43]. As a result, it is difficult to know how healthcare costs vary between responders and non-responders by BMI.

Overall, the effects estimated from the different analytical approaches were not significantly different. Economic evaluations typically include both mean values and their associated uncertainties in analyses. However, decisions stemming from these evaluations tend to use mean values. As a result, we would advise researchers to think carefully about cost inputs and to always incorporate uncertainty for transparently. Economic evaluations using the costs of obesity as inputs should be sex-specific and may benefit from incorporating ample parameter uncertainty in the model. More research is needed to understand why effect estimates vary between different analytical approaches.

### Electronic supplementary material

Below is the link to the electronic supplementary material.

Supplementary Material 1

Supplementary Material 2

## Footnotes

1 For example, if an individual moved from Norway in 2009 and moved back in 2010, and died in 2015 – costs for this individual were included for the years 2011–2015, and the average yearly costs were estimated as: costs 2011 + costs 2012 + costs 2013 + costs 2014 + costs 2015 / 5 years.

2 The World Health Organization (WHO) classifies obesity according to BMI. Overweight corresponds to BMI 25–30 kg/m^2^, class 1 obesity to BMI 30–35 kg/m^2^, and class 2 obesity to BMI 35–40 kg/m^2^.

3 Namsos, Steinkjer, Verdal, Levanger and Stjørdal.

4 4 All others includes: first-generation immigrants without a Norwegian background, individuals born in Norway by two foreign-born parents, foreign-born individuals with a Norwegian parent, Norwegian-born individuals with a foreign parent and individuals born abroad by Norwegian-born parents.

## Abbreviations

BMI: Body mass index
IV: Instrumental variable
OLS: Ordinary least squares
GLM: Generalized linear model
2PM: Two-part model
GRS: Genetic risk score
HUNT: The Trøndelag Health Studies
