# Supplementary material for: The healthcare costs of increased body mass index–evidence from The Trøndelag Health Study
Source: Health Econ Rev. 2024 Jun 1;14:36. doi: 10.1186/s13561-024-00512-8 (PMC11143647; doi:10.1186/s13561-024-00512-8)
Supplement: Supplementary file 1 — Supplementary Material 1 [file 13561_2024_512_MOESM1_ESM.docx]

S**UPPLEMENTARY INFORMATION**

**The healthcare costs of increased BMI – evidence from the HUNT study**

**List of Figures**

| **Figure S1** | Histogram comparing the main characteristics of the main sample and the three sub-samples used for the different methodological approaches, for males. | **p3** |
| --- | --- | --- |
| **Figure S2** | Histogram comparing the main characteristics of the main sample and the three sub-samples used for the different methodological approaches, for females. | **p4** |
| **Figure S3** | Coefficient values and 95% confidence interval for each covariate included in the different fully-adjusted analytical approaches – for males. | **p8** |
| **Figure S4** | Coefficient values and 95% confidence interval for each covariate included in the different fully-adjusted analytical approaches – for females. | **p9** |
| **Figure S5** | Estimated effect and 95% confidence intervals of BMI on healthcare costs for males and females when using each analytical approach on the main sample compared with when using the offspring sample. | **p10** |

**List of Tables**

| **Table S1** | Descriptive information about the sample used for the time-lagged models | **p5** |
| --- | --- | --- |
| **Table S2** | Descriptive information about the sample used for offspring analyses | **p6** |
| **Table S3** | Descriptive information about the sample used for the IV analyses with GRS instruments (Mendelian randomization) | **p7** |


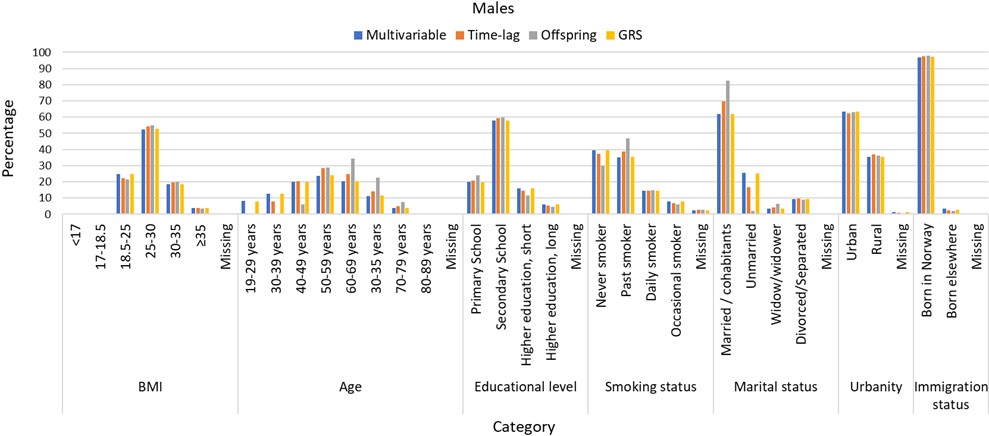


**Figure S1:** Histogram comparing the main characteristics of the main sample and the three sub-samples used for the different methodological approaches, for males.


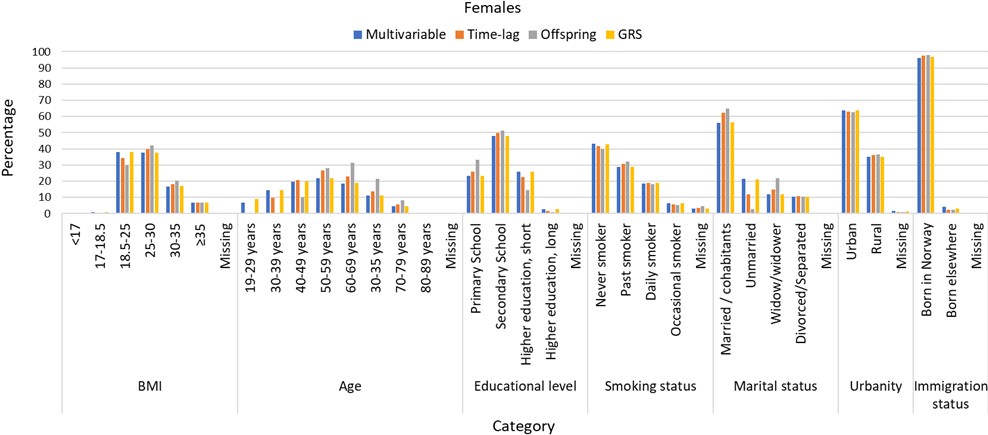


**Figure S2:** Histogram comparing the main characteristics of the main sample and the three sub-samples used for the different methodological approaches, for females.

**Table S1:** Descriptive information about the sample used for the time-lagged models.

| Variable | Category | Males n (%) | | Females n (%) | | Total n (%) | |
| --- | --- | --- | --- | --- | --- | --- | --- |
| Total | All | 17 589 | (100.0) | 21 119 | 100.0 | 50 039 | (100.0) |
| BMI- category^a^ | Underweight | 32 | (0.2) | 141 | (0.7) | 173 | (0.4) |
|  | Normal weight | 3 925 | (22.3) | 7 265 | (34.4) | 11 190 | (28.9) |
|  | Overweight | 9 535 | (54.2) | 8 453 | (40.0) | 17 988 | (46.5) |
|  | Class 1 obesity | 3 439 | (19.6) | 3 834 | (18.2) | 7 273 | (18.8) |
|  | Class 2 obesity | 658 | (3.7) | 1 426 | (6.8) | 2 084 | (5.4) |
| Age - category | 19-29 years | 2 | (0.0) | 5 | (0.0) | 7 | (0.0) |
|  | 30-39 years | 1 381 | (7.9) | 2 068 | (9.8) | 3 449 | (8.9) |
|  | 40-49 years | 3 548 | (20.2) | 4 374 | (20.7) | 7 922 | (20.5) |
|  | 50-59 years | 4 974 | (28.3) | 5 588 | (26.5) | 10 562 | (27.3) |
|  | 60-69 years | 4 328 | (24.6) | 4 864 | (23.0) | 9 192 | (23.7) |
|  | 70-79 years | 2 476 | (14.1) | 2 930 | (13.9) | 5 406 | (14.0) |
|  | 80-89 years | 841 | (4.8) | 1 197 | (5.7) | 2 038 | (5.3) |
|  | 90 + years | 39 | (0.2) | 93 | (0.4) | 132 | (0.3) |
|  | Missing | 0 | (0.0) | 0 | (0.0) | 0 | (0.0) |
| Educational level | Primary School | 3 621 | (20.6) | 5 467 | (25.9) | 9 088 | (23.5) |
|  | Secondary School | 10 457 | (59.5) | 10 513 | (49.8) | 20 970 | (54.2) |
|  | Higher education, short | 2 549 | (14.5) | 4 736 | (22.4) | 7 285 | (18.8) |
|  | Higher education, long | 941 | (5.3) | 367 | (1.7) | 1 308 | (3.4) |
|  | Missing | 21 | (0.1) | 36 | (0.2) | 57 | (0.1) |
| Smoking Status | Never smoker | 6 575 | (37.4) | 8 801 | (41.7) | 15 376 | (39.7) |
|  | Past smoker | 6 843 | (38.9) | 6 435 | (30.5) | 13 278 | (34.3) |
|  | Daily smoker | 2 537 | (14.4) | 3 977 | (18.8) | 6 514 | (16.8) |
|  | Occasional smoker | 1 177 | (6.7) | 1 176 | (5.6) | 2 353 | (6.1) |
|  | Missing | 457 | (2.6) | 730 | (3.5) | 1 187 | (3.1) |
| Marital status | Married / cohabitants | 12 229 | (69.5) | 13 175 | (62.4) | 25 404 | (65.6) |
|  | Unmarried | 2 923 | (16.6) | 2 514 | (11.9) | 5 437 | (14.0) |
|  | Widow/widower | 732 | (4.2) | 3 114 | (14.7) | 3 846 | (9.9) |
|  | Divorced/Separated | 1 697 | (9.6) | 2 302 | (10.9) | 3 999 | (10.3) |
|  | Missing | 8 | (0.0) | 14 | (0.1) | 22 | (0.1) |
| Urbanity | Urban | 10 982 | (62.4) | 13 286 | (62.9) | 24 268 | (62.7) |
|  | Rural | 6 478 | (36.8) | 7 643 | (36.2) | 14 121 | (36.5) |
|  | Missing | 129 | (0.7) | 190 | (0.9) | 319 | (0.8) |
| Immigration status | Born in Norway | 17 187 | (97.7) | 20 583 | (97.5) | 37 770 | (97.6) |
|  | Born elsewhere | 402 | (2.3) | 536 | (2.5) | 938 | (2.4) |
|  | Missing | 0 | (0.0) | 0 | (0.0) | 0 | (0.0) |

a=BMI-categories following WHO categorization: underweight (BMI<18.5 kg/m^2^), normal weight (BMI 18.5-25 kg/m^2^), overweight (BMI 25-30 kg/m^2^), class 1 obesity (BMI 30-35 kg/m^2^), and class 2 obesity (BMI 35-40 kg/m^2^).

**Table S2:** Descriptive information about the sample used for offspring analyses.

| **Variable** | **Category** | **Males** n (%) | | **Females** n (%) | | **Total** n (%) | |
| --- | --- | --- | --- | --- | --- | --- | --- |
| **Total** | All | 8 598 | (100.0) | 11 812 | 100.0 | 20 410 | (100.0) |
| **BMI- category^a^** | Underweight | 18 | (0.2) | 75 | (0.6) | 93 | (0.5) |
|  | Normal weight | 1 861 | (21.6) | 3 550 | (30.1) | 5 411 | (26.5) |
|  | Overweight | 4 713 | (54.8) | 4 957 | (42.0) | 9 670 | (47.4) |
|  | Class 1 obesity | 1 710 | (19.9) | 2 415 | (20.4) | 4 125 | (20.2) |
|  | Class 2 obesity | 296 | (3.4) | 815 | (6.9) | 1 111 | (5.4) |
| **Age - category** | 19-29 years | 0 | (0.0) | 0 | (0.0) | 0 | (0.0) |
|  | 30-39 years | 2 | (0.0) | 28 | (0.2) | 30 | (0.1) |
|  | 40-49 years | 523 | (6.1) | 1 190 | (10.1) | 1 713 | (8.4) |
|  | 50-59 years | 2 495 | (29.0) | 3 333 | (28.2) | 5 828 | (28.6) |
|  | 60-69 years | 2 941 | (34.2) | 3 712 | (31.4) | 6 653 | (32.6) |
|  | 70-79 years | 1 951 | (22.7) | 2 528 | (21.4) | 4 479 | (21.9) |
|  | 80-89 years | 652 | (7.6) | 957 | (8.1) | 1 609 | (7.9) |
|  | 90 + years | 34 | (0.4) | 64 | (0.5) | 98 | (0.5) |
|  | Missing | 0 | (0.0) | 0 | (0.0) | 0 | (0.0) |
| **Educational level** | Primary School | 2 055 | (23.9) | 3 941 | (33.4) | 5 996 | (29.4) |
|  | Secondary School | 5 133 | (59.7) | 6 051 | (51.2) | 11 184 | (54.8) |
|  | Higher education, short | 1 008 | (11.7) | 1 698 | (14.4) | 2 706 | (13.3) |
|  | Higher education, long | 393 | (4.6) | 99 | (0.8) | 492 | (2.4) |
|  | Missing | 9 | (0.1) | 23 | (0.2) | 32 | (0.2) |
| **Smoking Status** | Never smoker | 2 578 | (30.0) | 4 726 | (40.0) | 7 304 | (35.8) |
|  | Past smoker | 4 012 | (46.7) | 3 806 | (32.2) | 7 818 | (38.3) |
|  | Daily smoker | 1 261 | (14.7) | 2 125 | (18.0) | 3 386 | (16.6) |
|  | Occasional smoker | 503 | (5.9) | 623 | (5.3) | 1 126 | (5.5) |
|  | Missing | 244 | (2.8) | 532 | (4.5) | 776 | (3.8) |
| **Marital status** | Married / cohabitants | 7 089 | (82.4) | 7 676 | (65.0) | 14 765 | (72.3) |
|  | Unmarried | 182 | (2.1) | 311 | (2.6) | 493 | (2.4) |
|  | Widow/widower | 557 | (6.5) | 2 601 | (22.0) | 3 158 | (15.5) |
|  | Divorced/Separated | 766 | (8.9) | 1 219 | (10.3) | 1 985 | (9.7) |
|  | Missing | 4 | (0.0) | 5 | (0.0) | 9 | (0.0) |
| **Urbanity** | Urban | 5 438 | (63.2) | 7 403 | (62.7) | 12 841 | (62.9) |
|  | Rural | 3 106 | (36.1) | 4 318 | (36.6) | 7 424 | (36.4) |
|  | Missing | 54 | (0.6) | 91 | (0.8) | 145 | (0.7) |
| **Immigration status** | Born in Norway | 8 425 | (98.0) | 11 547 | (97.8) | 19 972 | (97.9) |
|  | Born elsewhere | 173 | (2.0) | 265 | (2.2) | 438 | (2.1) |
|  | Missing | 0 | (0.0) | 0 | (0.0) | 0 | (0.0) |

a=BMI-categories following WHO categorization: underweight (BMI<18.5 kg/m^2^), normal weight (BMI 18.5-25 kg/m^2^), overweight (BMI 25-30 kg/m^2^), class 1 obesity (BMI 30-35 kg/m^2^), and class 2 obesity (BMI 35-40 kg/m^2^).

**Table S3:** Descriptive information about the sample used for the IV analyses with GRS instruments (Mendelian randomization).

| Variable | Category | Males n (%) | | Females n (%) | | Total n (%) | |
| --- | --- | --- | --- | --- | --- | --- | --- |
| Total | All | 21 985 | (100.0) | 26 332 | 100.0 | 48 317 | (100.0) |
| BMI- category^a^ | Underweight | 61 | (0.3) | 221 | (0.8) | 282 | (0.6) |
|  | Normal weight | 5 455 | (24.8) | 9 980 | (37.9) | 15 435 | (31.9) |
|  | Overweight | 11 555 | (52.6) | 9 961 | (37.8) | 21 516 | (44.5) |
|  | Class 1 obesity | 4 104 | (18.7) | 4 437 | (16.9) | 8 541 | (17.7) |
|  | Class 2 obesity | 810 | (3.7) | 1 733 | (6.6) | 2 543 | (5.3) |
| Age - category | 19-29 years | 1 734 | (7.9) | 2 397 | (9.1) | 4 131 | (8.5) |
|  | 30-39 years | 2 749 | (12.5) | 3 785 | (14.4) | 6 534 | (13.5) |
|  | 40-49 years | 4 379 | (19.9) | 5 232 | (19.9) | 9 611 | (19.9) |
|  | 50-59 years | 5 255 | (23.9) | 5 790 | (22.0) | 11 045 | (22.9) |
|  | 60-69 years | 4 486 | (20.4) | 4 944 | (18.8) | 9 430 | (19.5) |
|  | 70-79 years | 2 520 | (11.5) | 2 939 | (11.2) | 5 459 | (11.3) |
|  | 80-89 years | 826 | (3.8) | 1 178 | (4.5) | 2 004 | (4.1) |
|  | 90 + years | 36 | (0.2) | 67 | (0.3) | 103 | (0.2) |
|  | Missing | 0 | (0.0) | 0 | (0.0) | 0 | (0.0) |
| Educational level | Primary School | 4 336 | (19.7) | 6 106 | (23.2) | 10 442 | (21.6) |
|  | Secondary School | 12 756 | (58.0) | 12 631 | (48.0) | 25 387 | (52.5) |
|  | Higher education. short | 3 525 | (16.0) | 6 806 | (25.8) | 10 331 | (21.4) |
|  | Higher education. long | 1 323 | (6.0) | 711 | (2.7) | 2 034 | (4.2) |
|  | Missing | 45 | (0.2) | 78 | (0.3) | 123 | (0.3) |
| Smoking Status | Never smoker | 8 711 | (39.6) | 11 302 | (42.9) | 20 013 | (41.4) |
|  | Past smoker | 7 766 | (35.3) | 7 603 | (28.9) | 15 369 | (31.8) |
|  | Daily smoker | 3 204 | (14.6) | 4 977 | (18.9) | 8 181 | (16.9) |
|  | Occasional smoker | 1 760 | (8.0) | 1 686 | (6.4) | 3 446 | (7.1) |
|  | Missing | 544 | (2.5) | 764 | (2.9) | 1 308 | (2.7) |
| Marital status | Married / cohabitants | 13 639 | (62.0) | 14 847 | (56.4) | 28 486 | (59.0) |
|  | Unmarried | 5 546 | (25.2) | 5 555 | (21.1) | 11 101 | (23.0) |
|  | Widow/widower | 741 | (3.4) | 3 119 | (11.8) | 3 860 | (8.0) |
|  | Divorced/Separated | 2 025 | (9.2) | 2 776 | (10.5) | 4 801 | (9.9) |
|  | Missing | 34 | (0.2) | 35 | (0.1) | 69 | (0.1) |
| Urbanity | Urban | 13 913 | (63.3) | 16 737 | (63.6) | 30 650 | (63.4) |
|  | Rural | 7 823 | (35.6) | 9 226 | (35.0) | 17 049 | (35.3) |
|  | Missing | 249 | (1.1) | 369 | (1.4) | 618 | (1.3) |
| Immigration status | Born in Norway | 21 380 | (97.2) | 25 523 | (96.9) | 46 903 | (97.1) |
|  | Born elsewhere | 605 | (2.8) | 809 | (3.1) | 1 414 | (2.9) |
|  | Missing | 0 | (0.0) | 0 | (0.0) | 0 | (0.0) |

a=BMI-categories following WHO categorization: underweight (BMI<18.5 kg/m^2^), normal weight (BMI 18.5-25 kg/m^2^), overweight (BMI 25-30 kg/m^2^), class 1 obesity (BMI 30-35 kg/m^2^), and class 2 obesity (BMI 35-40 kg/m^2^).


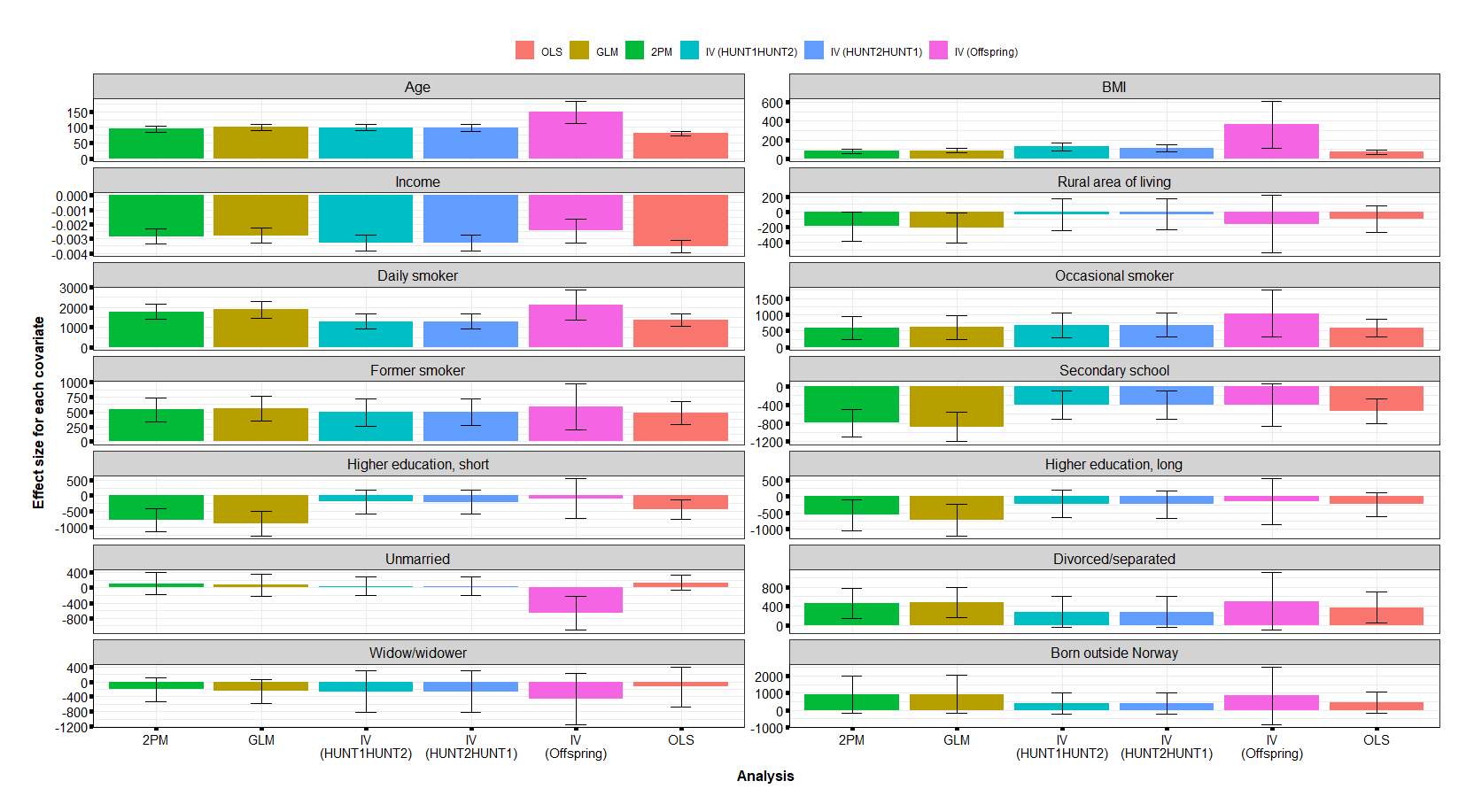
**Figure S3:** Coefficient values and 95% confidence interval for each covariate included in the different fully-adjusted analytical approaches – for males. The baseline values were: primary school (for educational level), never smoker (for smoking status), married/cohabitants (for marital status), urban area of living (for urbanity), and born in Norway (for country of birth).


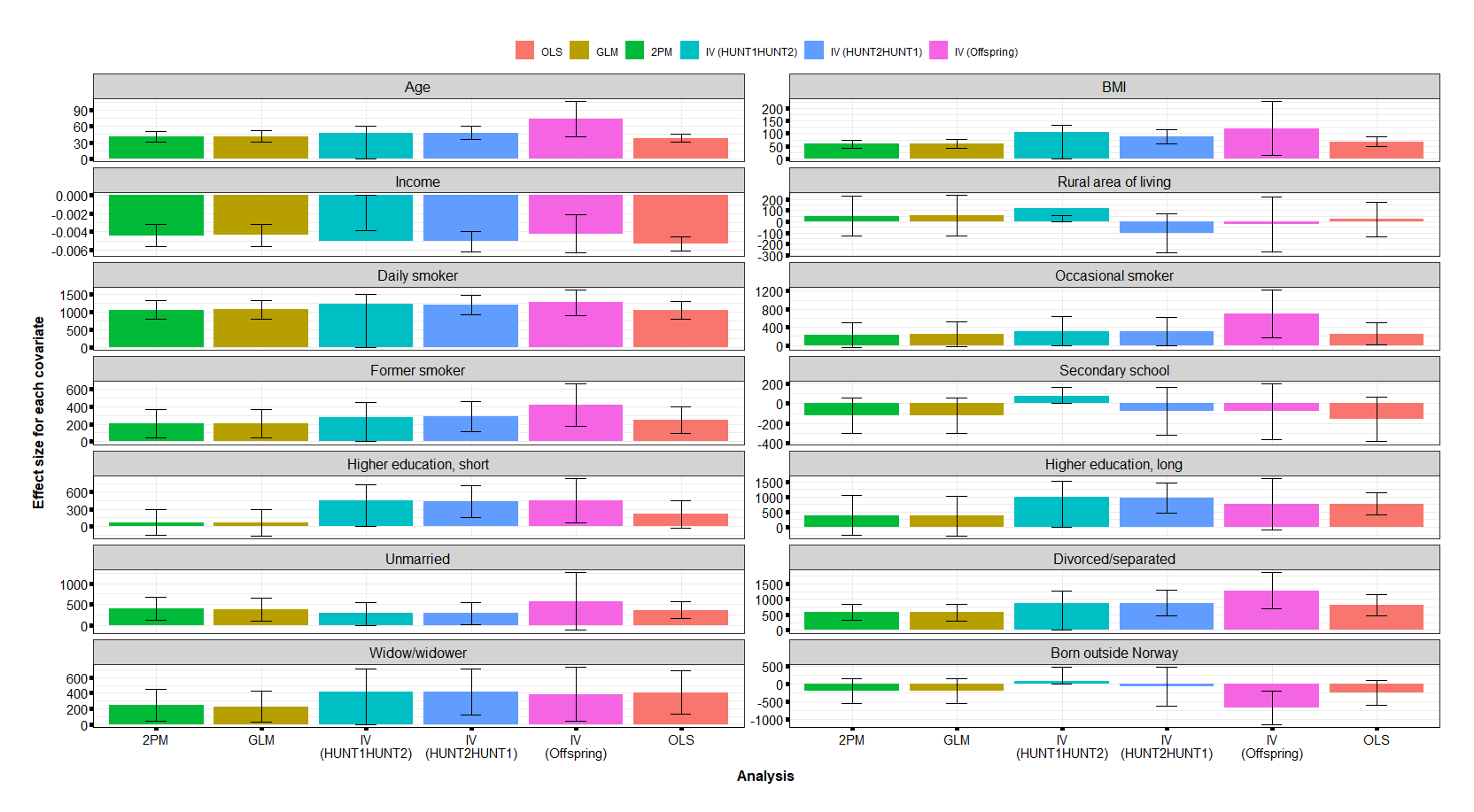


**Figure S4:** Coefficient values and 95% confidence interval for each covariate included in the different fully-adjusted analytical approaches – for females. The baseline values were: primary school (for educational level), never smoker (for smoking status), married/cohabitants (for marital status), urban area of living (for urbanity), and born in Norway (for country of birth).


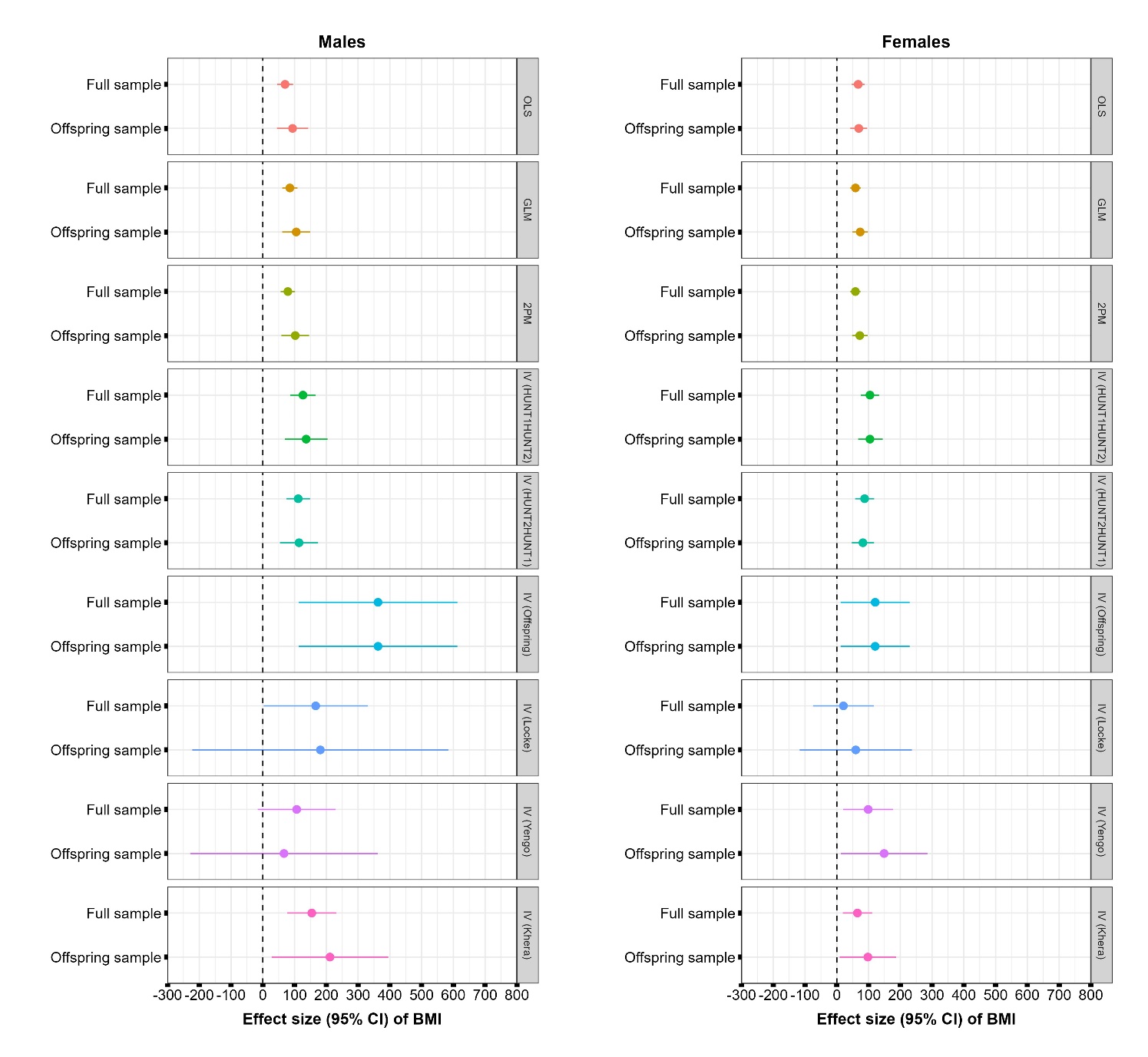


**Figure S5:** The estimated effect and 95% confidence intervals of BMI on healthcare costs for males (left) and females (right) when using each analytical approach on the main sample compared with when using the offspring sample.
